# Metal–Organic‐Framework‐Derived Carbon Nanostructures for Site‐Specific Dual‐Modality Photothermal/Photodynamic Thrombus Therapy

**DOI:** 10.1002/advs.201901378

**Published:** 2019-07-04

**Authors:** Fengrong Zhang, Yuehong Liu, Jiani Lei, Shunhao Wang, Xunming Ji, Huiyu Liu, Qi Yang

**Affiliations:** ^1^ Department of Radiology Xuanwu Hospital Beijing 100053 P. R. China; ^2^ Beijing Advanced Innovation Center for Soft Matter Science and Engineering State Key Laboratory of Organic‐Inorganic Composites Bionanomaterials & Translational Engineering Laboratory Beijing Key Laboratory of Bioprocess Beijing Laboratory of Biomedical Materials Beijing University of Chemical Technology Beijing 100029 P. R. China; ^3^ Department of Neurosurgery Xuanwu Hospital Beijing 100053 P. R. China

**Keywords:** carbon nanostructures, photodynamic therapy, photothermal therapy, thrombus

## Abstract

Although near‐infrared (NIR)‐light‐mediated photothermal thrombolysis has been investigated to overcome the bleeding risk of clinical clot‐busting agents, the secondary embolism of post‐phototherapy fragments (>10 µm) for small vessels should not be ignored in this process. In this study, dual‐modality photothermal/photodynamic thrombolysis is explored using targeting nanoagents with an emphasis on improving biosafety as well as ameliorating the thrombolytic effect. The nanoagents can actively target glycoprotein IIb/IIIa receptors on thrombus to initiate site‐specific thrombolysis by hyperthermia and reactive oxygen species under NIR laser irradiation. In comparison to single photothermal thrombolysis, an 87.9% higher re‐establishment rate of dual‐modality photothermal/photodynamic thrombolysis by one‐time treatment is achieved in a lower limb thrombosis model. The dual‐modality thrombolysis can also avoid re‐embolization after breaking fibrin into tiny fragments. All the results show that this strategy is a safe and validated protocol for thrombolysis, which fits the clinical translational trend of nanomedicine.

Thrombus associated diseases, such as ischemic stroke, acute myocardial infarction, and deep vein thrombosis, remain the leading causes of death or disability worldwide.^1^ Current clot‐busting agents with fibrinolytic drugs as the representatives are widely used in the clinic.^2^ However, the subsequent life‐threating bleeding risk with complicated physical monitoring represents a significant clinical limitation in this treatment.^3^

Near‐infrared (NIR)‐light‐mediated nano‐medicines provide a new therapeutic strategy for thrombus therapy via rapidly converting optical energy into hyperthermia by Landau damping effect.^4^ In 2016, Dash and co‐workers first demonstrated that NIR‐irradiated gold nanorods possessed antithrombotic properties for dissolving fibrin clots.^5^ After that, van Hest et al. reported a photothermal thrombolytic system based on Janus‐type erythrocyte membrane‐coated micromotors.^6^ These studies proved that photothermal therapy (PTT) could serve as an effective therapy for thrombolysis with specific spatiotemporal selectivity and minimal invasiveness.^7^ However, PTT might cause severe complications because the small blood vessels are possibly embolized by photothermal thrombolysis fragments, which are usually larger than 10 µm. Therefore, a more safe and effective therapy is intensely demanded.

Currently, reactive oxygen species (ROS)‐mediated photodynamic therapy (PDT) has been explored for peptide damages of fibrin biopolymers, e.g., polypeptide linkages, noncovalent interactions, and N‐attached biantennary glycan region, under the assistance of photosensitizers (PSs).^8^ Therefore, PDT might break the fibrin skeleton of blood clots and prevent secondary embolism from post‐photothermal fragments. However, most organic PSs molecules are still limited because of their lack of specific accumulation, poor resistance to photobleaching, and a short half‐life period in vivo.^9^ To minimize undesired effects and prevent secondary embolism, we thus hypothesized that an inorganic‐nanomaterial‐mediated dual‐modality photothermal/photodynamic strategy might present superior biosafety as well as thrombolytic effects.

Herein, we developed an Arg‐Gly‐Asp (RGD)‐modified mesoporous carbon nanospheres containing porphyrin‐like metal centers (RGD‐PMCS), which can initiate the site‐specific thrombolysis by hyperthermia and ROS under NIR laser irradiation. The therapeutic mechanism of phototherapy presented in **Scheme** 1 provides the following advantages: i) RGD‐PMCS could actively target the glycoprotein (GP) IIb/IIIa receptors on activated platelet surfaces,^10^ avoiding the high bleeding risk of the systematic fibrinolytic therapy.^11^ ii) ROS generated by RGD‐PMCS could damage the platelet factor 3 (PF3), a kind of lecithin, through lipid peroxidation and inhibit the thrombus recurrence.^12^ iii) The combination of photodynamic and photothermal therapy could greatly increase the efficiency of thrombus breaking and prevent the secondary embolism of larger fragments. On account of these features, this dual‐modality PTT/PDT provides a rapid, safe, and effective method for thrombolysis.

**Scheme 1:**
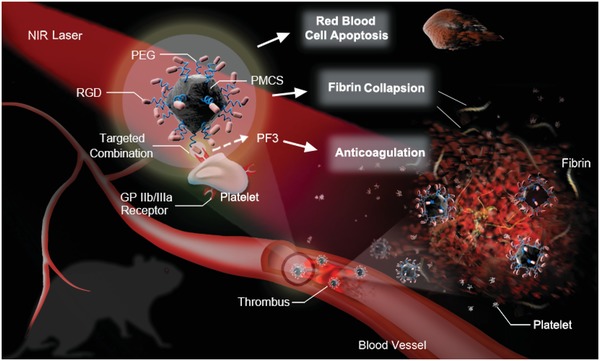
Schematic illustration of RGD‐PMCS‐mediated site‐specific photothermal/photodynamic thrombus therapy.

Monodisperse metal–organic framework (MOF)‐derived mesoporous carbon nanospheres containing porphyrin‐like metal centers (PMCS) were synthesized by carbonization of an imidazolate framework based on our previous reports (**Figure** 1a).^13^ The obtained nanostructures were further modified with Vitamin E–poly(ethylene glycol)–COOH (VE‐PEG‐COOH), then conjugated the RGD motifs via the formation of amide bonds. As shown in Figure 1b, the transmission electron microscopy (TEM) image revealed the PMCS with an overall size of about 120 nm. In addition, the hydrodynamic sizes of PMCS and RGD‐PMCS revealed by dynamic light scattering (DLS) were 220 and 295 nm, respectively (Figure 1c). The negative zeta potential of RGD‐PMCS is beneficial to increase the inter‐nanoparticles electrostatic repulsion, avoiding capillaries clogging (Figure 1d).^14^ Fourier transform infrared spectroscopy was adopted to characterize the chemical composition of PMCS as well as RGD‐PMCS and the existence of RGD motifs (Figure S1, Supporting Information). The −CH_2_− asymmetric and symmetric stretching vibration of RGD‐PMCS had double bands at 2852 and 2928 cm^−1^, and the characteristic peak (1628 cm^‐1^) was attributed to the stretching vibration of C=O. In addition, the peak at 3190 cm^‐1^ reflected –NH_2_ groups, demonstrating the existence of RGD peptides. Element mapping and energy‐dispersive X‐ray spectroscopy (EDS) spectra of PMCS and RGD‐PMCS indicated that carbon, oxygen, nitrogen, and zinc were homogeneously distributed in the nanoparticles (Figures S2,S3, Supporting Information).

**Figure 1:**
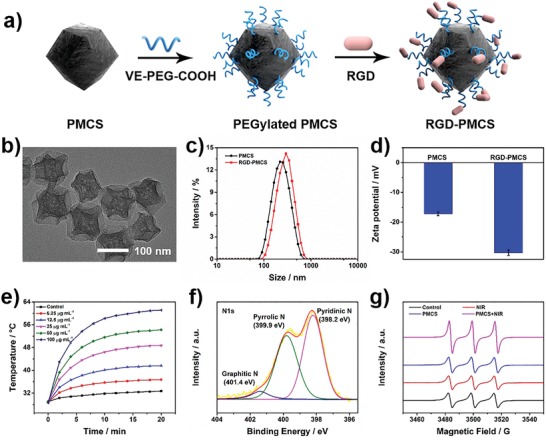
Characterization of PMCS and RGD‐PMCS. a) Scheme for RGD‐PMCS synthesis. b) TEM image of PMCS. c) Size distribution and d) zeta potentials of PMCS and RGD‐PMCS. e) Temperature elevation of PMCS with different concentrations (0, 6.25, 12.5, 25, 50, and 100 µg mL^‐1^) under 808 nm laser irradiation (2 W cm^‐2^, 20 min). f) N1s XPS spectra for PMCS. g) ESR spectra of ^1^O_2_ by PMCS (100 µg mL^‐1^) with or without NIR laser irradiation (808 nm, 2 W cm^‐2^, 3 min).

NIR region absorption is indispensable for subsequent dual‐modality PTT/PDT. The ultraviolet–visible–NIR (UV–vis–NIR) absorption spectrum for PMCS clearly indicated absorption across the spectral range from 300 to 1000 nm, revealing the crucial potential for NIR‐mediated phototherapy (Figure S4, Supporting Information). And the elevated temperature of PMCS aqueous dispersions with NIR laser irradiation showed the significant concentration‐ and time‐dependency (Figure 1e, and Figure S5a, Supporting Information). For the PMCS under NIR light irradiation, when the concentration reached up to 100 µg mL^−1^, the temperature boosted to 61.1 °C within 20 min. Furthermore, the photothermal conversion efficiency (η) of PMCS was determined to be 33.3% and the extinction coefficient was calculated to be 6.35 L g^−1^ cm^−1^, which was higher than that of many other reported photothermal nanomaterials (Figure S5b, Supporting Information).^15^ The high‐resolution X‐ray photoelectron spectroscopy (XPS) for N1s chemical states were conducted, three remarkable peaks at 398.2, 399.9, and 401.4 eV were assigned to pyridinic, pyrrolic, and graphitic N, respectively (Figure 1f). The special zinc‐centered porphyrin (a kind of photosensitizer)‐like structure in PMCS and the similar coordination number to porphyrin Zn suggested PMCS contains catalytic centers to generate ROS.[qv: 13b] To further explore the intrinsic photodynamic characteristics of PMCS, we measured the singlet oxygen (^1^O_2_, a kind of ROS) produced by PMCS under NIR laser irradiation with electron spin resonance (ESR) spectroscopy. Compared with other groups, the signal intensity of ^1^O_2_ generated by PMCS with 808 nm laser irradiation showed an approximate onefold increase (Figure 1g). These results implied that PMCS had excellent photosensitivity, which provided enormous potential in PTT and PDT for thrombus treatment.

It is well known that activated platelets contact with the subendothelial extracellular matrix at the site of endothelial damage, allowing platelets aggregate and bind to the fibrinogen in plasma with high affinity, causing the formation of thrombus.^16^ Therefore, as shown in **Figure** 2a, platelets were assessed by flow cytometry with the assistance of fluorescein isothiocyanate‐CD41 (FITC‐CD41) for predicting whether the PSs would trigger platelets activation. Platelets activated with adenosine diphosphate (ADP) were adopted as a positive control.^17^ Conversely, platelets without any treatment were explored as a negative control. Based on the results after co‐culturing resting platelets with RGD‐PMCS or irradiating by NIR laser, there was nearly no change in comparison with the negative control. In addition, the potential cytotoxicity of PMCS and RGD‐PMCS were assessed via measuring human umbilical vein endothelial cells (HUVEC) viability after co‐culturing for 24 h. There was nearly no significant apoptosis or necrosis was observed when the concentration of PMCS and RGD‐PMCS ranged from 6.25 to 100 µg mL^−1^ (Figure 2b). The hemolysis rate of RGD‐PMCS with a series of concentrations was much lower than the upper limited value (5%), confirming excellent biosafety of RGD‐PMCS (Figure 2c).

**Figure 2:**
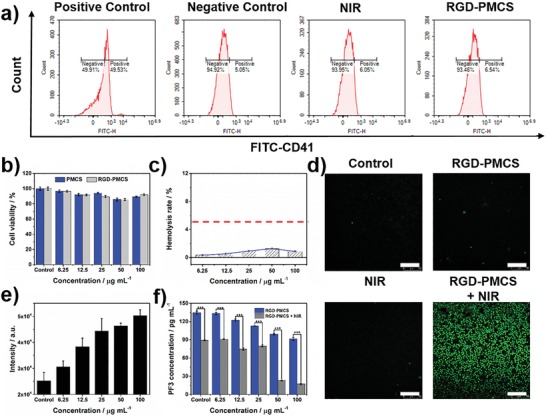
a) Flow cytometry quantification platelets after co‐culturing with RGD‐PMCS (100 µg mL^‐1^) or irradiating by NIR laser (808 nm, 2 W cm^−2^, 3 min). ADP activated platelets were adopted as positive group. Platelets without any treatment were explored as negative control. b) Relative viabilities of HUVEC after co‐incubation with different concentrations of PMCS or RGD‐PMCS. c) Hemolysis rate of RGD‐PMCS. d) The intracellular ROS production of erythrocytes co‐incubated with RGD‐PMCS at 100 µg mL^−1^ under NIR laser irradiation (808 nm, 2 W cm^−2^, 3 min). Scale bar = 100 µm. e) Quantitative analysis ROS generation in erythrocytes after treating with various concentrations of RGD‐PMCS combined 808 nm light (2 W cm^−2^, 3 min). f) Level of PF3 after various treatments. *P* > 0.05, *P < 0.05, **P < 0.01, ***P < 0.001.

Next, to investigate the therapeutic mechanism of the generated ROS by NIR laser irradiation combined with RGD‐PMCS, the photodynamic effect of RGD‐PMCS + NIR was explored. As indicated in Figure 2d, the generation of ROS was detected by 2,7‐dichlorodihydrofluorescein diacetate probe. Notably, nearly no fluorescence could be discerned in RGD‐PMCS‐treated erythrocytes. By comparison, the significant fluorescence signal was presented after exposing to the NIR laser due to the large production of ROS. Then, we further quantitatively analyzed and proved that the production of ROS increased with nanoparticles concentration‐dependency, (Figure 2e). Moreover, accumulated studies have indicated that excessive ROS could produce significant lipid peroxidation on phospholipids in cells.^18^ Thus, we hypothesized that ROS induced by RGD‐PMCS under NIR laser irradiation could damage PF3. As expected, after exposing to an 808 nm laser, the amount of PF3 dramatically decreased with the increase of RGD‐PMCS concentration. Furthermore, RGD‐PMCS solution at 100 µg mL^−1^ induced about 82.5% PF3 impair, which could provide potential anticoagulant therapy to inhibit re‐embolism of blood vessels (Figure 2f).

Encouraged by the satisfactory photothermal and photodynamic performances of RGD‐PMCS under NIR laser irradiation, we further conducted an FITC‐labeled fibrin clots assay, an intuitive approach for assessing the structural fission of the fibrin skeleton. Importantly, opposed to the green‐fluorescence‐labeled fibrin without any treatment, the large amount of the collapsed skeleton and small pieces were presented after co‐culturing fibrin with RGD‐PMCS and exposing with NIR laser, which indicated that the fiber skeleton was broken by the action of thermal energy and oxidation of ROS (**Figure** 3a). To certify this result, artificial thrombosis model produced by thrombin and vein blood was further established. The absorbance of the supernatant was examined at 450 and 540 nm as indicators of fibrin and hemoglobin. After incubation with RGD‐PMCS and irradiation with an 808 nm laser, the photothermal and photodynamic ablation process of the clot was monitored. And the fibrin absorption curve was prominently increased after 4 min irradiation, suggesting the synergistic effect of PTT/PDT on thrombus with subsequent disintegration (Figure 3b,c). Additionally, the hydrodynamic size of the released fragments after the treatment revealed by DLS was ≈1.1 µm, which could be eventually filtered through the anticlogging system of glomerular filtration membranes to avoid embolism in the cardiovascular system (Figure S6, Supporting Information).^19^ Considering that the erythrocytes are the constituent parts of the thrombus, their apoptosis is indispensable for clot thorough disintegration. So hemolysis analysis induced by PTT/PDT of RGD‐PMCS was performed. As shown in Figure 3d, the hemolysis rate of erythrocytes dramatically boosted with the increasing RGD‐PMCS and 100 µg mL^−1^ RGD‐PMCS solution resulted in 30% cells ruptured under 808 nm laser irradiation. This result was additionally certified with the help of calcein‐AM, a fluorescent probe for indicating living cells. Erythrocytes in NIR and RGD‐PMCS groups showed high viability, and no apparent apoptosis was discerned. By comparison, when combined NIR laser with nanomaterials, the intracellular fluorescence signal decreased rapidly, illustrating the effective erythrocytes killing ability of RGD‐PMCS (Figure S7, Supporting Information).

**Figure 3:**
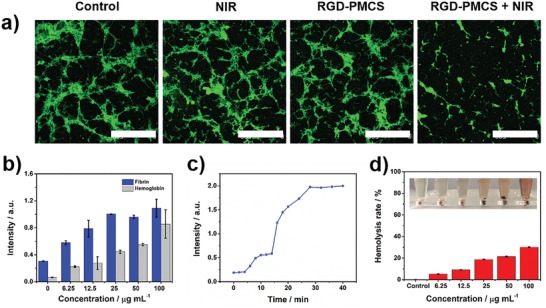
a) Confocal laser scanning microscopy (CLSM) images of fibrin clots after incubation with 100 µg mL^‐1^ RGD‐PMCS with or without NIR laser irradiation (808 nm, 2 W cm^−2^, 3 min). Scale bar = 500 µm. b) Level of fibrin and hemoglobin in artificial blood clot supernatant incubated with various concentrations of RGD‐PMCS and irradiated by an 808 nm laser (2 W cm^−2^, 20 min). c) Fibrin levels in artificial blood clot supernatant after treatment with RGD‐PMCS (100 µg mL^−1^) and NIR laser (808 nm, 2 W cm^−2^) at different time points. d) Hemolysis rate of RGD‐PMCS at different concentrations after irradiated by an 808 nm laser (2 W cm^−2^, 3 min). The inset shows the corresponding photograph of hemolysis. The group irradiated by the laser without RGD‐PMCS was adopted as a control.

Thrombus‐specific targeting strategy of RGD‐PMCS was based on the expressed GPIIb/IIIa receptors on activated platelets (**Figure** 4a). In the initiatial platelets aggregation process, the multiple adhesive ligands, such as von Willebrand factor (VWF) and exposed collagen on damaged subendothelial matrix, bind the integrins on activated platelets, including GPIb‐IX‐V and GPVI receptors. Besides, the subsequent propagation of thrombi is driven by agonists released from the platelets, including ADP and thromboxane A2 (TXA2). And the fibrinogens combine the activated GPIIb/IIIa receptors to cross‐link the activated platelets, which increase the density of the thrombus.^20^ Herein, RGD‐PMCS could competitively combine the activated GPIIb/IIIa receptors to avoid further thrombus formation. To quantitatively evaluate the targeting capabilities of the designed nanoparticles, inductively coupled plasma mass spectrometry was used to detect the Zn concentration on activated platelets after co‐culturing with PMCS and RGD‐PMCS. Compared with the PMCS group, a significant higher quantify of Zn (4.2 µg mL^−1^) was detected in the RGD‐conjugated particles, thereby allowing for greater in vivo targeting therapy (Figure S8, Supporting Information). Carbon‐based nanoparticles have been investigated as photoacoustic (PA) contrast agents with high tissue penetration and excellent spatial resolution due to high NIR absorption. Here, we identified the PA signals of PMCS and RGD‐PMCS in lower limb thrombus model to evaluate the specific target ability of RGD. On account of the effective RGD‐PMCS accumulation at the thrombotic site, the signals especially reached a maximum at 1 h post intravenous injection, approximately 5 h faster than that of PMCS, demonstrating its great potential to effectively shorten the treatment time for thrombolysis (Figure S9, Supporting Information). Subsequently, inspired by the positive synergistic effects of RGD‐PMCS‐mediated PDT/PTT in vitro, we further verified the in vivo therapeutic effects for lower limb blood vessel of rats. First, the changes of local temperature at the thrombotic site under NIR light irradiation were monitored by an infrared thermal imager. It was found that the thrombotic temperature in phosphate buffered saline (PBS) injection group and PMCS + NIR group increased about 5.9 and 10.5 °C, respectively, while the temperature treated with RGD‐PMCS under 808 nm laser irradiation increased about 14.6 °C, confirming effective accumulation of RGD‐PMCS and the possibility of in vivo thrombus destruction via the local hyperthermia (Figure 4b, and Figure S10, Supporting Information). For discovering the therapeutic effects in vivo, the rats were randomly divided into seven groups: 1) normal group; 2) embolized group; 3) PMCS group; 4) RGD‐PMCS group; 5) NIR group; 6) PMCS + NIR group; 7) RGD‐PMCS + NIR group. Histopathology assessments showed that nearly no thrombus in the blood vessels in the RGD‐PMCS + NIR group after the one‐time treatment (Figure 4c). Additionally, magnetic resonance imaging (MRI) verified the embolism and recanalization of the treated vessels in vivo, which displayed an obvious lateral vascular recanalization signal in the synergistic treatment group (Figure 4d). The regional blood signal intensity of the RGD‐PMCS + NIR group was 2.7‐fold and 7.7‐fold than that of the PMCS + NIR group and RGD‐PMCS group, and achieving relatively high thrombolytic effect with re‐establishment ratio up to 87.9% compared with the normal blood vessels (Figure S11, Supporting Information).

**Figure 4:**
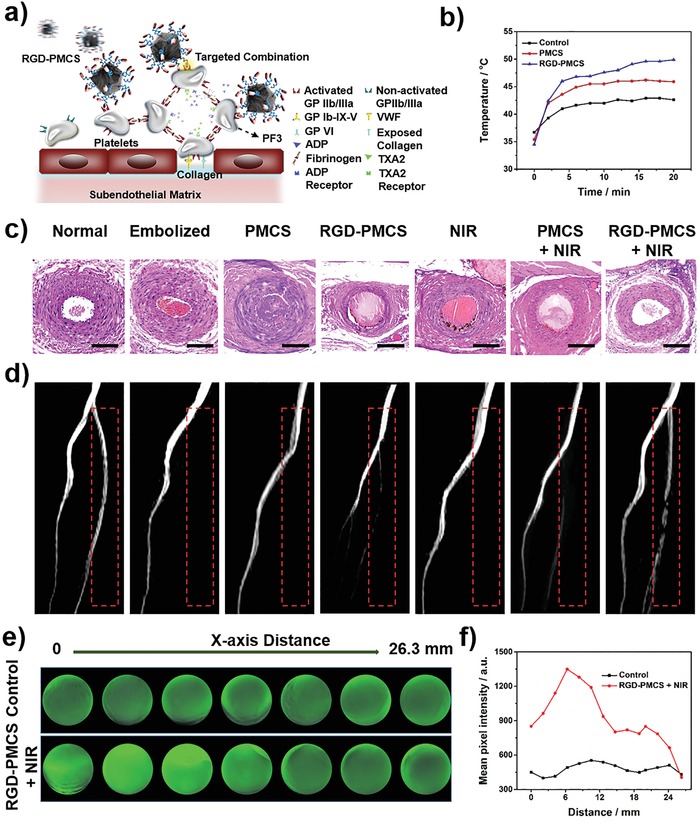
a) Scheme for site‐specific targeting thrombus of RGD‐PMCS. b) Temperature increase curves of left lower limb of rats intravenously injected with PBS, PMCS (10 mg kg^‐1^), and RGD‐PMCS (10 mg kg^‐1^) and irradiated by NIR laser (2 W cm^−2^, 20 min). c) Histopathology and d) MRI of rats after various treatments. Scale bar = 100 µm. The red dashed line indicates the site of thrombus. e) PA images and f) Quantitative analysis of the signals collected from simulated blood vessel in different cross sections after different treatments (2 W cm^‐2^, 10 min).

The mice treated with PMCS and RGD‐PMCS showed there was no obvious body weight loss during the observation period (Figure S12, Supporting Information). And no side effect was detected in major tissue slices, indicating that negligible toxicity of the treatment group, which was consistent with the hematology analysis (Figure 2c, and Figure S13, Supporting Information). To analyze the biodistribution of nanoparticles in vivo, healthy BALB/c mice were injected with fluorescent dye indocyanine green‐conjugated RGD‐PMCS. The results showed that accompanied with the extension of the nanoparticles circulation time, the fluorescence signal of major organs gradually decreased, indicating part of particles could be metabolized from the body (Figure S14, Supporting Information).

Anticoagulant ability of PDT induced by PF3 peroxidation damage was further evaluated via the mouse tail tip transection model, which has been extensively used to analyze the anticoagulant effect of mice that administrated with antiplatelet drugs. Mice treated with RGD‐PMCS + NIR showed 2.4‐fold and 1.5‐fold longer bleeding time compared with the control group and PMCS + NIR group, proving the anticoagulant potential of PDT (Figure S15, Supporting Information).

Research on PTT has shown that thermal energy could increase the tissue penetration of nanomaterials.^21^ So we assumed that the energy could also promote RGD‐PMCS into the deeper site of thrombus via local photothermal effect. To evaluate the penetration efficacy of RGD‐PMCS cooperated with NIR laser irradiation, the simulated blood vessel was established. And the PA imaging was used to observe the position of the contrast agents. As illustrated in Figure 4e,f, the clot of the synergistic group displayed a crucial signal enhancement with a penetration of about 26.3 mm, suggesting the designed PTT/PDT synergistic therapy could not only remove the thrombus and produce a specific antiplatelet effect by hyperthermia and ROS, but also enhanced the penetration of the nanomaterials.

In summary, we have demonstrated the resultant RGD‐PMCS exhibited satisfactory thrombus‐targeting ability to the GPIIb/IIIa receptors on activated platelets at thrombus sites. With the enhanced accumulation of RGD‐PMCS and shortened therapeutic time, the dual‐modality PTT/PDT systematic therapy mediated by RGD‐PMCS could be efficiently utilized for thrombolysis without secondary embolism. In addition, PDT‐induced high‐efficiency ROS could damage the PF3 by lipid peroxidation, thereby avoiding re‐embolism. Overall, the great biosafety and effectiveness suggest that this dual‐modality therapeutic strategy holds a promising future for thrombus therapy.

## Experimental Section

Experimental details and additional characterization can be found in the Supporting Information. All the experiments and animal procedures were conducted in accordance with the Guidelines of the Animal Experiments and Experimental Animals Management Committee of Capital Medical University. The study protocol was approved by the Animal Experiments and Experimental Animal Welfare Committee of Capital Medical University (Permit Number: AEEI‐2017‐117).

## Conflict of Interest

The authors declare no conflict of interest.

## Supporting information

Supplementary
