# Supplementary material for: Metal–Organic‐Framework‐Derived Carbon Nanostructures for Site‐Specific Dual‐Modality Photothermal/Photodynamic Thrombus Therapy
Source: Adv Sci (Weinh). 2019 Jul 4;6(17):1901378. doi: 10.1002/advs.201901378 (PMC6724354; doi:10.1002/advs.201901378)
Supplement: Supplementary file 1 — Supplementary [file ADVS-6-1901378-s001.pdf]

Copyright WILEY-VCH Verlag GmbH & Co. KGaA, 69469 Weinheim, Germany, 2019.

ADVANCED SCIENCE
Open Access

Supporting Information

for *Adv. Sci.,* DOI: 10.1002/advs.201901378

Metal–Organic-Framework-Derived Carbon Nanostructures for Site-Specific Dual-Modality Photothermal/Photodynamic Thrombus Therapy

Fengrong Zhang, Yuehong Liu, Jiani Lei, Shunhao Wang, Xunming Ji, Huiyu Liu, and Qi Yang**

Copyright WILEY-VCH Verlag GmbH & Co. KGaA, 69469 Weinheim, Germany, 2019.

Supporting Information

Metal–Organic-Framework-Derived Carbon Nanostructures for Site-Specific Dual-Modality Photothermal/Photodynamic Thrombus Therapy

Fengrong Zhang, Yuehong Liu, Jiani Lei, Shunhao Wang, Xunming Ji, Huiyu Liu, and Qi Yang**

Experimental Section

Materials. Zinc nitrate hexahydrate ($Zn(NO_3)_2{\cdot}6H_2O$), cetyltrimethylammonium bromide (CTAB), tetraethyl orthosilicate (TEOS), calcium chloride ($CaCl_2$), and 2-methylimidazole were obtained from Macklin Biochemical Technology Co., Ltd. (Shanghai, China). Vitamin E-polyethylene glycol-COOH (VE-PEG-COOH, MW = 5000) was purchased from Shanghai Ponsure Biotech, Inc. (Shanghai, China). Indocyanine green (ICG) and sodium citrate were purchased from Sigma, St. Louis (MO, USA). N-hydroxysuccinimide (NHS) and 1-ethyl-3(3-dimethylaminopropyl) carbodiimide hydrochloride (EDC) were purchased from Adamas-beta Corporation (Shanghai, China). Arg-Gly-Asp (RGD) motifs were obtained from GL Bio-chem Ltd. (Shanghai, China). Fluorescein isothiocyanate (FITC)-CD41 (FITC-CD41) was purchased from Biolegend (CA, USA). FITC-conjugated fibrinogen was purchased from Abcam (MA, UK). Adenosine diphosphate (ADP), 3-(4,5-Dimethylthiazol-2-yl)-2,5-diphenyltetrazolium bromide (MTT), bicinchoninic acid (BCA) assay kit, calcein acetoxymethyl ester (calcein-AM), and 2,7-dichlo-rodihydrofluorescein diacetate (DCFH-DA) were obtained from Beijing

Solarbio Science & Technology Co., Ltd. (Beijing, China). Rat platelet factor 3 (PF3) enzyme-linked immunosorbent assay (ELISA) kits were purchased from j&l Biological Industrial Co., Ltd. (Shanghai, China). Hematoxylin and eosin (H&E) were obtained from Boster Biological Technology Co., Ltd. (Wuhan, China). Deionized water was obtained from a Milli-Q synthesis system. All chemicals were used as received without further treatment.

Nanoparticle Characterization. A HT-7700 transmission electron microscope (TEM) was used to characterize the morphology and structure of metal–organic framework–derived mesoporous carbon nanospheres containing porphyrin-like metal centers (PMCS). Element mapping and energy-dispersive X-ray spectroscopy (EDS) spectrum of PMCS and RGD motifs modified PMCS (RGD-PMCS) were performed by APOLLO XLT2 TEM. Zeta potentials and the hydrodynamic size of PMCS and RGD-PMCS were analyzed by Zetasizer Nano-ZS (Malvern Instruments, UK). Fourier transform infrared spectra were acquired with a Nicolet 6700 spectrometer by using the KBr pellet method. The ultraviolet-visible-infrared absorption spectrum was obtained by 2600 UV-visible Spectrophotometer (SHIMADZU, Japan). An infrared thermal imager (FLIR SC 620; FLIR system, Inc., Wilsonville, OR, USA) was used to record the photothermal images. And the temperature was acquired with a thermocouple (TES 1315, TES Electrical Electronic Corp., Taiwan, China). Porphyrin-like structure in the PMCS was analyzed by ESCALab220i-XL high-performance electron spectrometer. Singlet oxygen ($^1O_2$) production was quantified by using Bruker ELEXSYS E500 CW electron spin resonance (ESR)

spectrometer.

Preparation of PMCS. PMCS were prepared by carbonization of an imidazolate framework according to our earlier reports.[1,2] Firstly, 33.75 mmol 2-methylimidazole dissolved in 100 mL methanol was poured into a solution containing 8 mmol $Zn(NO_3)_2 \cdot 6H_2O$ in (100 mL), and then stirred at room temperature for 3 h. The obtained zeolitic-imidazolate-framework (ZIF-8) nanoparticles were collected by centrifugation and washed three times with methanol. Later, ZIF-8 nanoparticles were dispersed in 240 mL 10 vol% methanol, and the mixture was adjusted to pH 11 by NaOH aqueous, and 0.2 g CTAB was further added. After stirring at 500 rpm for 30 min, 1.2 mL TEOS was added into the above solution, followed by stirring for another 30 min. The obtained ZIF-8 core/mesoporous silica shell (ZIF-8@mSiO_2) nanoparticles collected by centrifugation and washed three times with ethanol and then dried at 60 °C. Next, the prepared ZIF-8@mSiO_2 nanoparticles were pyrolyzed at 800 °C for 2 h with the help of flowing N_2, and then cooled to room temperature. Then, the pyrolyzed sample was etched by 4 M NaOH solution at 80 °C for 3 h. The final product was collected by centrifugation and washed several times with ultrapure water until the value of pH was close to neutral.

Preparation of RGD-PMCS. Firstly, 2 mg PMCS were mixed with 2 mg VE-PEG-COOH in 3 mL of ultrapure water. After vibrating at 37 °C for 4 h, the precipitates were washed three times with deionized water and collected after 10-min centrifugation at 12000 rpm. Then, 3 mL deionized water was supplemented, and the pH was adjusted to 6.0 with hydrochloric acid. Subsequently, 58 mM NHS (200 μL)

and 200 mM EDC (200 μL) were added. After vibrating for 1 h at 37 °C, 2 mg RGD peptide was added into the mixture. After 3 h, the solution was centrifuged at 15000 rpm for 10 min and the precipitate was washed three times. The residual RGD concentration in the supernatant was measured by the BCA protein assay. The conjugation efficiency of RGD (%) = (RGD input – free RGD in the supernatant)/RGD input × 100%. The final conjugation efficiency of RGD was 28.5%.

Preparation of ICG-Conjugated RGD-PMCS (ICG-RGD-PMCS). The prepared RGD-PMCS were resuspended in 3 mL ultrapure water and adjust the pH value to 6.0 with hydrochloric acid. Subsequently, NHS (58 mM, 200 μL) and EDC (200 mM, 200 μL) were added and vibrated for 1 h at 37 °C, and ICG was further added. The mixture was then vibrated at 4 °C overnight, and ICG-RGD-PMCS were obtained by centrifugation again.

In Vitro Photothermal Measurement. A series of PMCS aqueous dispersions (1 mL) with different concentrations (0, 6.25, 12.5, 25, 50, 100 μg mL^{-1}) were irradiated with near-infrared (NIR) laser (2 W cm^{-2}) for 20 min, and the temperature change was recorded by a thermocouple and the photothermal images were acquired by an infrared thermal imager.

$^1O_2$ Measurements by ESR. Production of $^1O_2$ by PMCS (100 μg mL^{-1}) with or without NIR irradiation (808 nm, 2 W cm^{-2}, 3 min) was evaluated by ESR spectrometer with 2,2,6,6-tetramethylpiperide (TEMP) as the trapping probe.

Platelets and Red Blood Cells Preparation. Sprague Dawley (SD) rats, male,

weighted 250-300 g, were purchased from Beijing Vital River Laboratory Animal Technology Co. Ltd and all the experiments and animal procedures were conducted in accordance with the Guidelines of the Animal Experiments and Experimental Animals Management Committee of Capital Medical University. The study protocol was approved by the Animal Experiments and Experimental Animal Welfare Committee of Capital Medical University (Permit Number: AEEI-2017-117). Blood was drawn from the inferior vein of healthy rats under anesthesia and collected in heparinized tubes with 4% sodium citrate (1 mL) to avoid coagulation. For experiments involving washed platelets, the whole blood was centrifuged at 800 rpm for 8 min. The platelet-rich plasma (PRP) in the supernatant was further diluted by 5 mL Tyrode's solution and centrifuged at 3500 rpm for 10 min and the precipitate was washed by Tyrode's solution for three times. For experiments with washed red blood cells, the whole blood was separated by centrifugation at 2000 rpm for 10 min, and then washed three times with phosphate buffered saline (PBS).

In Vitro Blood Biosafety Evaluation. To demonstrate that the photosensitizers could not activate platelets in the blood, the prepared platelets were diluted with Tyrode's solution with a density of 1×10^7 per milliliter and divided into four groups: a) Positive control group, b) Negative control group, c) NIR group (808 nm, 2 W cm^{-2}, 3 min), d) RGD-PMCS group (100 μg mL^{-1}). For the positive control group, the platelets were activated with 10 μM ADP (250 μL) at 37 °C for 10 min. After adding 5 mM $CaCl_2$ (50 μL) and 50 μg mL^{-1} FITC-CD41 (10 μL), all groups were incubated at room temperature for 1 h and evaluated via flow cytometry. For hemolysis evaluation,

the collected red blood cells were diluted to 1/10 of their volume with PBS. Then 200 μL RGD-PMCS with different concentrations were added into 1.3 mL freshly isolated blood and then kept at 37 °C for 4 h, followed by centrifugation at 3000 rpm for 10 min. After that, the supernatant was collected and measured absorbance at 540 nm. PBS was acted as the negative control and deionized water was adopted as the positive control. Hemolysis ratio was calculated by the following formula: hemolysis % = (sample absorbance − negative control absorbance)/(positive control absorbance − negative control absorbance) × 100%.

In Vitro Cell Cytotoxicity Test. The human umbilical vein endothelial cells (HUVEC) were obtained from Peking Union Medical College Hospital (Peking, China). Fetal bovine serum (FBS), Ham's F12K, heparin, endothelial cell growth supplement (ECGS), L-glutamine, penicillin, and streptomycin were obtained from Corning (New York, USA). The cells were maintained in Ham's F12K medium with heparin (0.1 mg mL^{-1}), ECGS (0.05 mg mL^{-1}), 10% FBS, 1% L-glutamine and 1% penicillin/streptomycin at 37 °C containing 5% CO_2. For in vitro cytotoxicity test, HUVECs were seeded into 96 well plates with a density of 1×10^4 per well. After 24 h, cells were incubated with RGD-PMCS for another 24 h. The MTT assay was conducted following the standard protocol.

In Vitro Synergistic Therapy. Red blood cells were incubated with 100 μg mL^{-1} RGD-PMCS for 24 h. Subsequently, the cells were irradiated with an 808 nm laser at a power intensity of 2 W cm^{-2} for 3 min, and cultured for another 24 h. Then, the cells were stained with calcein-AM, followed by a Leica SP5 confocal laser scanning

microscope (CLSM) observation. Quantitative analysis was also taken followed the operation as the above treatments. Then the red blood cells were stained with DCFH-DA for 30 min and evaluated through a BioTek Powerwave XS fluorescence microplate reader.

For hemolysis treatment analysis, the diluted red blood cells were incubated with various concentrations of RGD-PMCS, followed by irradiating with the 808 nm laser (2 W cm^{-2}) for 3 min. After that, all operations were the same as the hemolysis evaluation described above.

Level of PF3 Detection. The prepared platelets were diluted with Tyrode's solution with a density of 1×10^{12} per milliliter and then treated with various concentrations of RGD-PMCS. After 1 h incubation, those platelets were subjected to irradiation of NIR laser (808 nm, 2 W cm^{-2}, 3 min). Then, the platelets were seeded into 96-well plates and kept at 37 °C for 30 min. Subsequently, the excess solution was removed and washed with detergent for 5 times, the platelets were incubated with enzyme standard reagent, incubated and washed in the same manner as described above. In addition, platelets were further incubated with Reagent A and then with Reagent B according to the protocol. After that, the reaction was stopped with Stop solution and measured absorbance at 540 nm.

FITC-Labeled Fibrin Clots Assay. FITC-labeled fibrin clots were induced by the addition of 25 U mL^{-1} thrombin and 2.5 mM $CaCl_2$ into a fibrinogen solution containing 1 mg mL^{-1} fibrinogen (450 μL) and 1 mg mL^{-1} FITC-labeled fibrinogen (50 μL), followed by incubation at 37 °C for 1 h. The colt was incubated with

RGD-PMCS (100 μg mL^{-1}, 500 μL) for 1 h and further irradiated with 808 nm laser (2 W cm^{-2}, 3 min) and evaluated through a CLSM.

In Vitro Target Specificity. The prepared activated platelets were diluted with Tyrode's solution and incubated with 100 μg mL^{-1} PMCS and RGD-PMCS for 1 h. The platelets were then washed three times with Tyrode's solution, and 200 μL aqua regia was added, the amount of Zn was measured by inductively coupled plasma mass spectrometry (X Series 2; ThermoFisher, Waltham, MA, USA).

Artificial Thrombosis Assay. Inferior vein blood was collected from healthy rats under anesthesia. Artificial thrombosis was produced by adding 25 U mL^{-1} thrombin and 25 mM $CaCl_2$ at 37 °C for 3 h. The resulted clot was incubated with different concentrations of RGD-PMCS for 1h and further exposed to 808 nm irradiation for 20 min at a power density of 2 W cm^{-2}, the absorbance at 540 nm and 340 nm were monitored. In addition, the hydrodynamic size of the artificial thrombosis supernatant was monitored at 10-min intervals by dynamic light scattering.

Lower Limb Thrombosis Model. SD rats (male, weighted 250-300 g) were anesthetized with chloral hydrate. For each of rat, the left lower limb was fixed after the pre-operation skin preparation, and then an incision was made to expose the targeting lower limb artery. One piece of filter paper saturated with 10% $FeCl_3$ was placed on the top of the left lower limb artery for 10 min to generate vascular injury. As for the mice (BALB/c, male, aged 6-8 weeks) thrombosis model, the lower limb artery was dealt with 5% $FeCl_3$ for 10 min.

In Vivo PA Imaging. The mice with lower limb thrombosis were intravenously

injected with PMCS (10 mg kg^{-1}) and RGD-PMCS (10 mg kg^{-1}), respectively, and photoacoustic (PA) images were collected by real-time multispectral optoacoustic tomography (MSOT inVision 128, iThera medical, Germany).

In Vivo Infrared Thermal Imaging. The rats with lower limb thrombosis were intravenously injected with PBS, PMCS (10 mg kg^{-1}) and RGD-PMCS (10 mg kg^{-1}), respectively. The thrombotic site was irradiated by NIR light (2 W cm^{-2}) for 20 min after 1 h post-injection, and the changes of temperature at the thrombotic site were monitored by an infrared thermal imager.

In Vivo Thrombosis Therapy. The rats with thrombosis were randomly divided into seven groups: 1) Normal group; 2) Embolized group; 3) PMCS group (10 mg kg^{-1}); 4) RGD-PMCS group (10 mg kg^{-1}); 5) NIR group (2 W cm^{-2}, 20 min); 6) PMCS + NIR group (10 mg kg^{-1}, 2 W cm^{-2}, 20 min); 7) RGD-PMCS + NIR group (10 mg kg^{-1}, 2 W cm^{-2}, 20 min). All of the treatments carried out only once. The H&E of major organs (heart, liver, spleen, lung, and kidney) and blood vessels were performed 1 day after treatments to assess the biosafety and thrombolytic effect of RGD-PMCS. Body weights of mice were recorded every three days.

Magnetic Resonance Imaging. During the Magnetic Resonance Imaging (MRI) scanning, rats in each group were continually sedated by chloral hydrate. All images were obtained from a 7-Tesla MRI scanner (Bruker, Germany). The experimental groups were shortly imaged after thrombus induction with the body coil of rats (Bruker, Germany).

After the scanning of the localizer, a 2-dimensional, time-of-flight magnetic

resonance angiogram was performed from the level of iliac bifurcation to the terminal lower limbs blood vessels. Sequence parameters were as follows: repetition time/echo time = 18/4.5 ms, number of averages = 3, and excitation pulse angle = 76°. With a 256 × 256 matrix, a field of view of 55 × 55 mm was scanned with 100 continuous slices of 600 μm thickness. In addition, the saturated zone was used to reduce the impacts of breath and lightly move to image quality. We also took some measures to protect rats from damages of low body temperature before and after scanning. The total scanning time was about 18 min.

The post-processed angiographic images, including 3-dimensional reconstructed images with the maximum-intensity project algorithm were made by Horos (version 3.3.5). The lower limb blood vessels of various groups were equally divided into three segments. Then, the quantitative analysis was provided in each segment by calculating the MRI signal area ratio, which was defined in accordance with the following equation: (Signal Area of Experimental Blood Vessel/Signal Area of Normal Blood Vessel) × 100%.

In Vivo Biodistribution. The healthy mice were injected via the tail with ICG-RGD-PMCS (10 mg kg^{-1}) (three mice per group). Then, the mice were sacrificed and major organs (heart, liver, spleen, lung, and kidney) were collected at 6, 12, 24, and 48 h post-injection. The fluorescence intensity organs were measured via using an IVIS Spectrum Imaging System (PerkinElmer, Waltham, MA, USA).

Tail Bleeding Assay. The mice with thrombosis were divided randomly into seven groups with the same operations as in vivo thrombosis therapy described above. A

distal segment with 10 mm of the tail was cut by anatomical scissors and the injured tail was put into 37 °C saline. The bleeding time was defined as the time needed for a wound to hemostasis for at least 10 s.

Intra-Thrombosis Penetration Analysis. Blood collected from the inferior vein of healthy rats was injected into a plastic pipette and the other end was closed. Artificial thrombosis was produced by adding 25 U mL^{-1} (100 μL) thrombin and 25 mM $CaCl_2$ at 37 °C for 3 h. The resulted clot was incubated with RGD-PMCS (1 mg mL^{-1}, 1 mL) and further exposed to 808 nm irradiation for 10 min at a power density of 2 W cm^{-2}, the PA signals pre- and post-treatment with RGD-PMCS were recorded by MSOT.

Statistical Analysis. All data were presented as the mean ± standard deviation or mean. Statistical analysis was conducted by using t-test: ns: $P > 0.05$, *$P < 0.05$, **$P < 0.01$, ***$P < 0.001$.

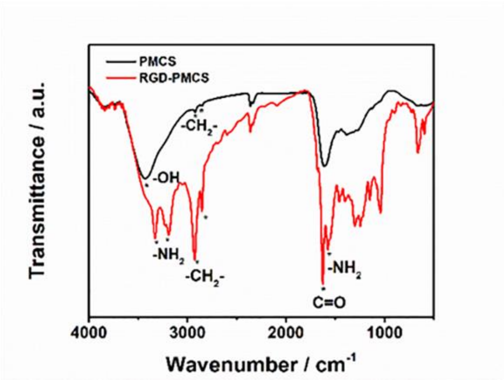

Figure S1. Fourier transform infrared spectra of PMCS and RGD-PMCS.

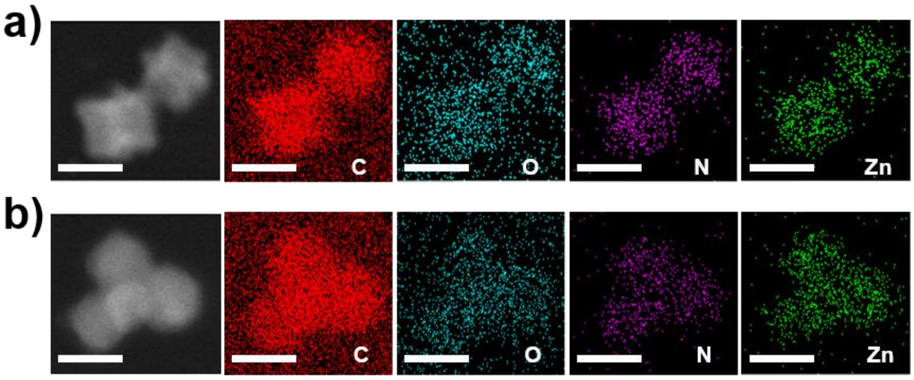

Figure S2. Elemental mapping for a) PMCS and b) RGD-PMCS. Scale bar = 100 nm.

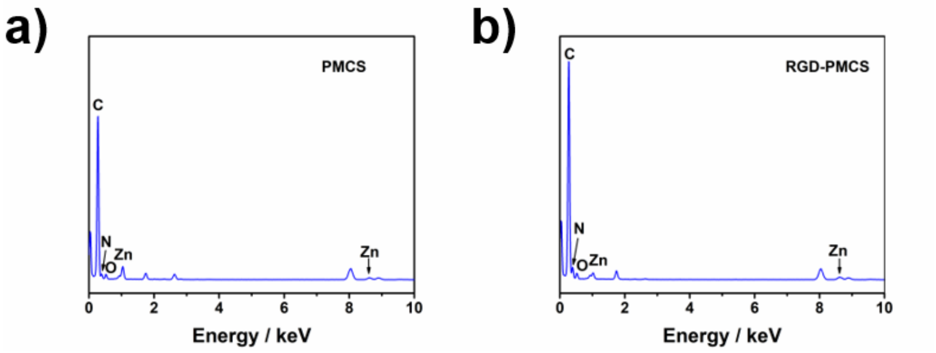

Figure S3. Energy-dispersive X-ray spectra of a) PMCS and b) RGD-PMCS.

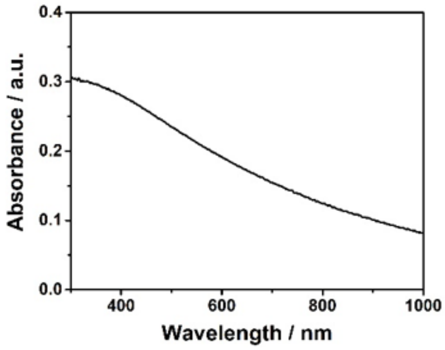

Figure S4. UV–vis–NIR absorption spectrum of PMCS.

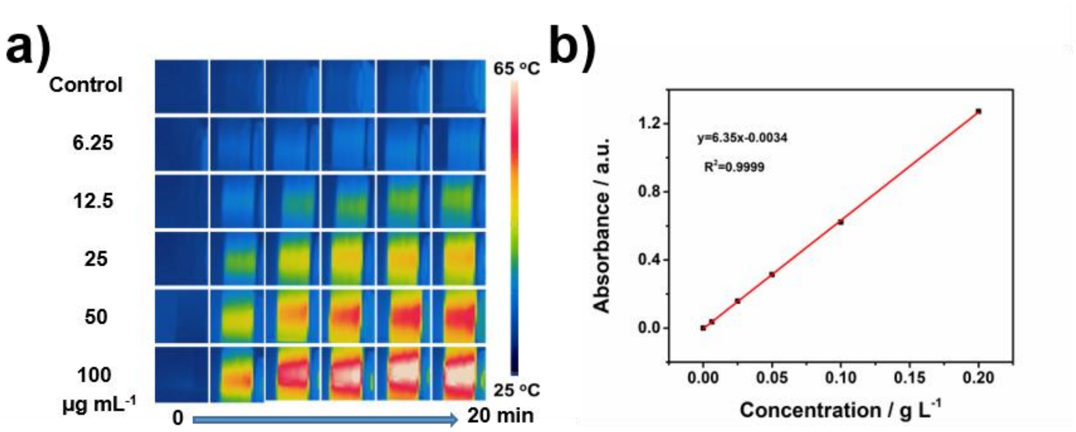

Figure S5. a) Infrared thermal images of PMCS at different concentrations under an 808 nm laser irradiation (2 W cm^{-2}, 20 min). b) Fitting curve of the absorption values at 808 nm of PMCS dispersions with different concentrations in water.

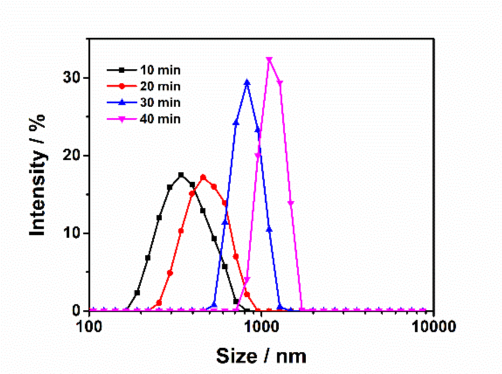

Figure S6. Hydrodynamic size of the released fragments treated with RGD-PMCS (100 μg mL^{-1}) and NIR laser (808 nm, 2 W cm^{-2}) at different time points.

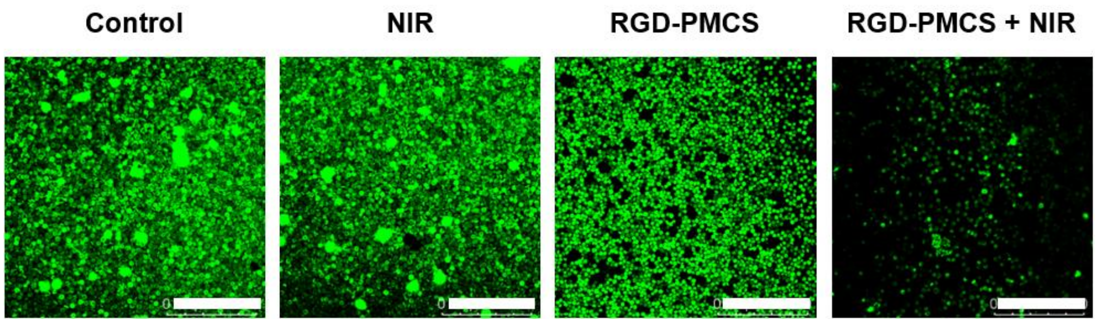

Figure S7. CLSM images of red blood cells stained with calcein-AM after co-incubating with RGD-PMCS (100 μg mL^{-1}) and irradiating with NIR laser (808 nm, 2 W cm^{-2}, 3 min). Scale bar = 100 μm.

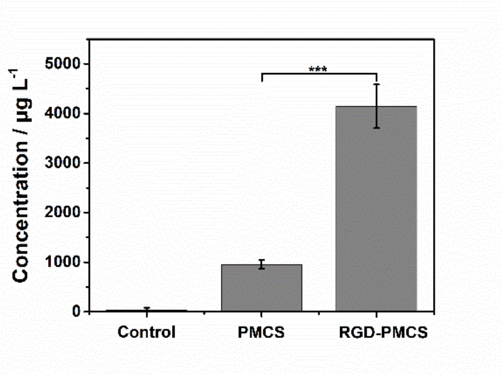

Figure S8. Quantitative analysis of Zn concentration in platelets after incubation with PMCS and RGD-PMCS. $P > 0.05$, *$P < 0.05$, **$P < 0.01$, ***$P < 0.001$.

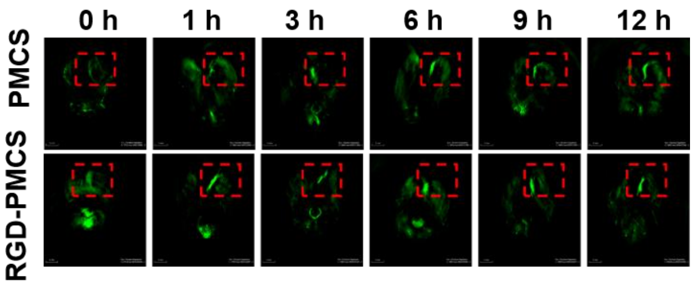

Figure S9. PA imaging of mice after intravenous injection of PMCS (10 mg kg^{-1}) and RGD-PMCS (10 mg kg^{-1}).

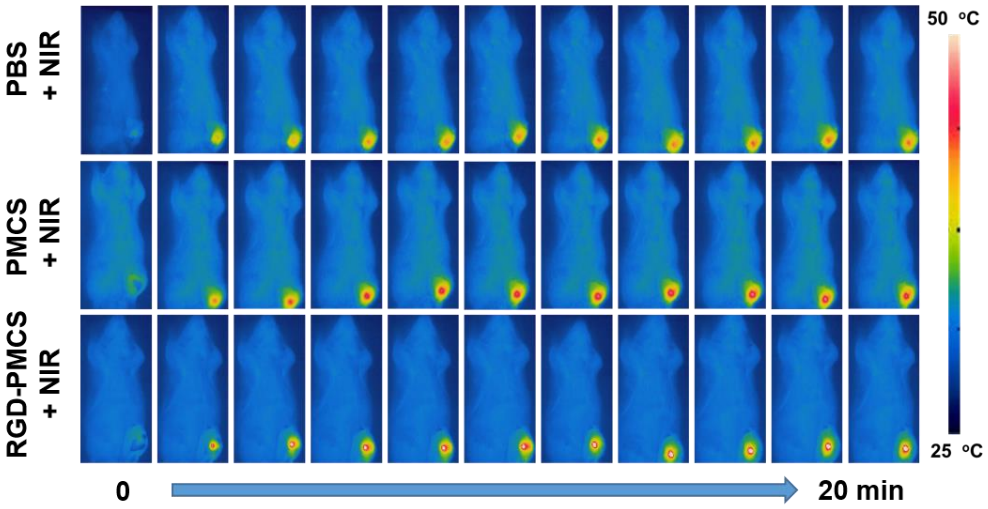

Figure S10. Infrared thermal images of rats intravenously injected with PBS, PMCS (10 mg kg^{-1}), and RGD-PMCS (10 mg kg^{-1}) and irradiated with NIR laser (2 W cm^{-2}, 20 min).

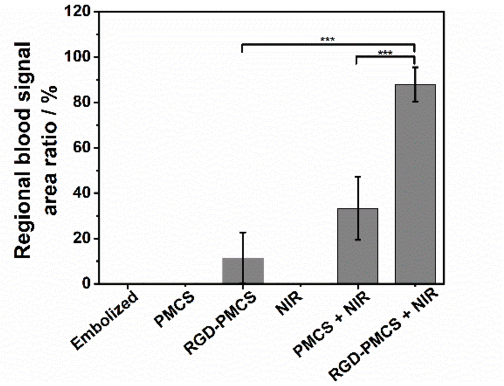

Figure S11. Quantitative analysis of MRI signal area ratio between the thrombotic vessels after various treatments and normal blood vessels. $P > 0.05$, *$P < 0.05$, **$P < 0.01$, ***$P < 0.001$.

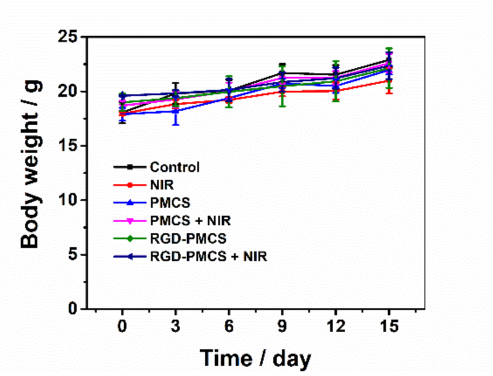

Figure S12. Body weights of variation of mice in different groups after various treatments.

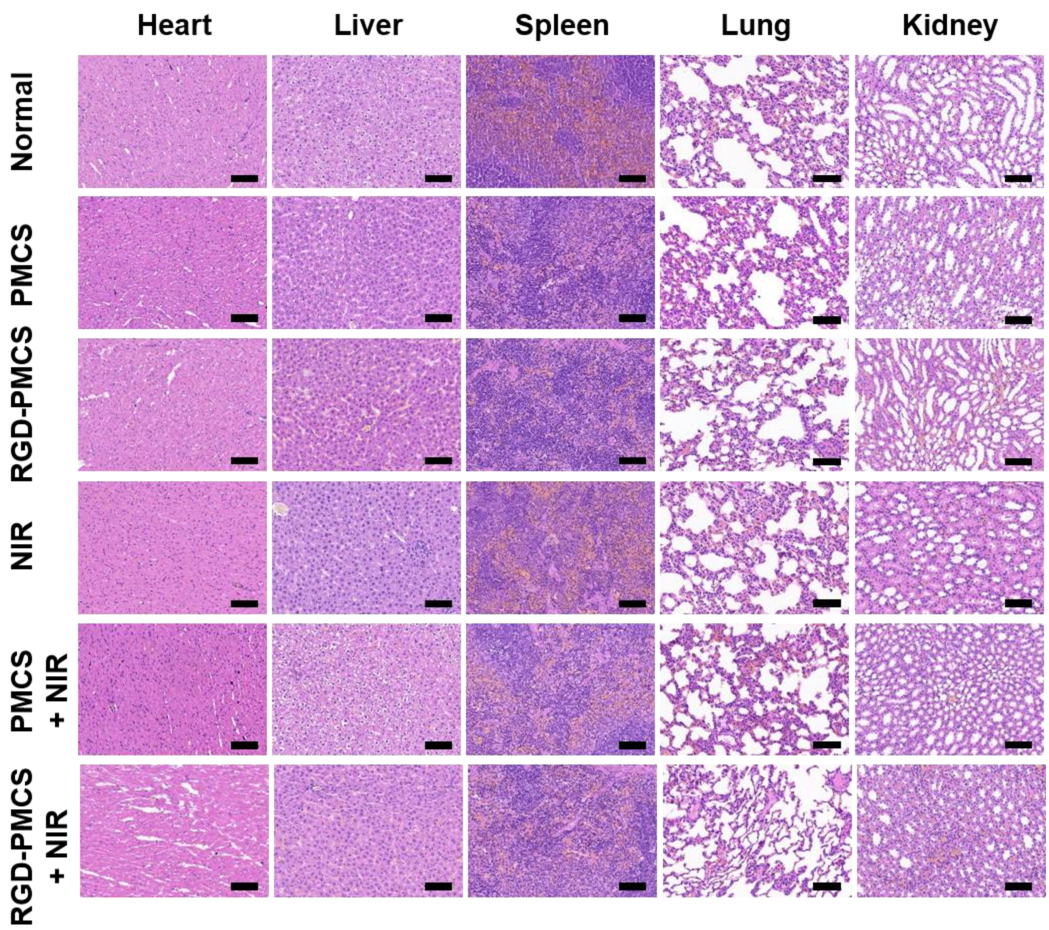

Figure S13. H&E stained images of major organs after various treatments. Scale bar = 100 μm.

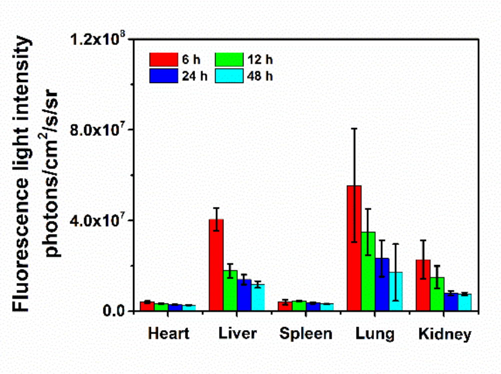

Figure S14. Quantitative analysis of fluorescence in major organs collected from mice with RGD-PMCS injection at different time points.

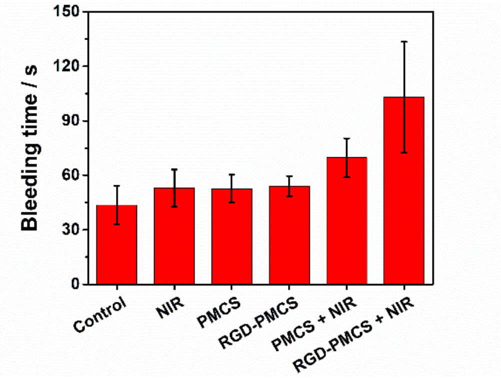

Figure S15. Bleeding time of the mice transected tail with different treatments.

References

[1] X. Pan, L. Bai, H. Wang, Q. Wu, H. Wang, S. Liu, B. Xu, X. Shi, H. Liu, *Adv. Mater.* **2018**, *30*, 1800180.

[2] S. Wang, L. Shang, L. Li, Y. Yu, C. Chi, K. Wang, J. Zhang, R. Shi, H. Shen, G. I. N. Waterhouse, S. Liu, J. Tian, T. Zhang, H. Liu, *Adv*. *Mater*. **2016**, *28*, 8379.
